# Rapport building and witness memory: Actions may ‘speak’ louder than words

**DOI:** 10.1371/journal.pone.0256084

**Published:** 2021-08-13

**Authors:** Zacharia Nahouli, Coral J. Dando, Jay-Marie Mackenzie, Andreas Aresti

**Affiliations:** 1 Department of Psychology, University of Westminster, London, United Kingdom; 2 Department of Criminology, University of Westminster, London, United Kingdom; University of Birmingham, UNITED KINGDOM

## Abstract

Building rapport during police interviews is argued as important for improving on the completeness and accuracy of information provided by witnesses and victims. However, little experimental research has clearly operationalised rapport and investigated the impact of rapport behaviours on episodic memory. Eighty adults watched a video of a mock crime event and 24-hours later were randomly allocated to an interview condition where verbal and/or behavioural (non-verbal) rapport techniques were manipulated. Memorial performance measures revealed significantly more correct information, without a concomitant increase in errors, was elicited when behavioural rapport was present, a superiority effect found in both the free and probed recall phase of interviews. The presence of verbal rapport was found to reduce recall accuracy in the free recall phase of interviews. Post-interview feedback revealed significant multivariate effects for the presence of behavioural (only) rapport and combined (behavioural + verbal) rapport. Participants rated their interview experience far more positively when these types of rapport were present compared to when verbal (only) rapport or no rapport was present. These findings add weight to the importance of rapport in supporting eyewitness cognition, highlighting the potential consequences of impoverished social behaviours for building rapport during dyadic interactions, suggesting ‘doing’ rather than simply ‘saying’ may be more beneficial.

## Introduction

There is widespread agreement that rapport building is an effective skill for improving interpersonal communication [1–4]. For example, in therapeutic settings rapport can support the development of positive relationships, resulting in improved goal outcomes (e.g., curbing maladaptive behaviours [5–8]). In retail, rapport between employees and customers is known to improve satisfaction and customer loyalty [9]. In collaborative learning contexts, rapport has been found to enhance understanding of cognitive goals which supports learners to feel more comfortable, allowing them to target cognitive resources on learning [10].

Rapport is also seen as vital in forensic interview settings for improving the ‘quality’ of an interview, which can increase the amount of information elicited from both persons of interest and witnesses/victims of crime (from hereon we use the term witness to include both; see [11–15]). In the case of witnesses, which is the focus of this research, various types of rapport have been found to improve the amount of information recalled and reduce errors of commission [16]. Researchers have also found that rapport can have an inoculating effect on witness memory, apparently reducing the negative impact of post-event misinformation [17–20]. Forensic interviews with witnesses are widely acknowledged as being socially and cognitively demanding and, in such contexts, effective communication is important. Here, key assumptions are centred on arguments that rapport has the potential to relieve some of the social demands of an interview, with a view to increasing capacity for cognitive processes such as episodic remembering [21–26]. Further, it has been argued that a comfortable witness may be more compliant [20, 27]. The corollary being that witnesses are more likely to work harder to recall an event resulting in a more complete and accurate account, including the revelation of more investigative-relevant information (e.g., [28–30]).

Despite a consensus as to the importance of rapport in forensic interviews, it is a challenging interpersonal behaviour to define, which necessarily constrains efforts to investigate the impact of rapport on witness memory at retrieval. Rapport is often variously and abstractly described across domains, such as being a “harmonious, empathetic, or sympathetic relation or connection” [31], “a smooth, positive interpersonal interaction” ([32], p. 208), or “a relationship marked by conformity” ([33], p. 51). A focused review of the forensic rapport literature also reveals a large research focus on i) mapping the presence and/or absence of rapport-building techniques and their likely impact on interview outcomes (e.g., [25, 26, 34]), or ii) developing and applying models of rapport and interpersonal communication using real-life interview data (e.g., [11, 28, 35–37]). Some focus has been placed on experimentally manipulating the presence/absence of various rapport behaviours in witness interview settings, although this research base is currently small and limited [14, 15]. However, understanding the impact of rapport on episodic memory performance during witness interviews is important and has real potential to guide interviewers, particularly at the start of an interviewing career for example.

To date, few studies have experimentally manipulated individual rapport behaviours, in part because of a lack of consensus in terms of operationalising rapport variables, but also because controlling potentially confounding variables such as interviewer variability is challenging [15]. Additionally, the empirical findings of experimental research that has manipulated rapport techniques with mock witnesses is mixed to a degree, again in part because of different approaches and methods. Some research has shown that building rapport has a positive effect on witness memory recall [16, 20, 38, 39], whereas others have found negative or inconsequential effects [18, 40]. Accordingly, in a witness interview context, numerous empirical questions have yet to be fully explored, including whether different types of rapport behaviours impact on witness memory, revealing itself in differential performance for example, and how various rapport behaviours are received by adult witnesses [15]. Towards filling this gap in understanding rapport-based communication on memory, we report an experimental study investigating several operationalised rapport-building techniques during interviews with adult mock witnesses by analysing memory performance and collecting post-interview feedback.

### Rapport in witness interviews

Witness memories are not objective recordings of an event, but fragile and unique personal records of a specific event (see [41]). Reconstructing and recounting these experiences, or episodes, is akin to mental time travel (see [13, 42, 43]), which requires concentration and cognitive effort on the part of the witness [44]. Police interviewers are tasked with managing the social context of a witness interview to maximise the amount of information recalled without inducing errors [23]. In doing so they have to clearly explain the interview process and the memory retrieval techniques as the interview progresses, and ensure witnesses feel physically comfortable and understand what is being asked of them. This requires effective communication, which many argue necessarily includes rapport building to form a ‘connection’ or a ‘relationship’ with a witness [16].

Rapport is an interpersonal behaviour comprising numerous techniques for developing and maintaining a comfortable social environment and enhancing communication [45, 46]. However, the empirical literature base is minimal and because rapport in witness interview contexts is so poorly operationalised it is challenging to understand *what* rapport-building techniques are used, and *how* and *when* they are used. This makes unpacking the impact of rapport-building techniques difficult [14, 15, 47]. For example, the cognitive interview advocates the importance of empathic behaviour and personalising the interview [23], while others emphasise active listening [46], being attentive and friendly [16], being open, interested and approachable [29], or some simply describe rapport as a ‘connection’ [48].

One conceptualisation of rapport that is often referred to in forensic interview contexts is the Tickle-Degnen and Rosenthal model [4], which suggests that rapport is established through the culmination of three interrelated components: mutual *positivity* (1) and *attention* (2), which leads to *coordination* (3) whereby the interaction becomes fluid and natural. In establishing and maintaining rapport, Tickle-Degnen and Rosenthal emphasise the importance of non-verbal behaviours such as smiling, nodding, directing body and eye gaze towards the interviewee, and having an open posture. Elaborations to the Tickle-Degnen and Rosenthal model have been made to include verbal behaviours. For example, Abbe and Brandon highlight verbal active listening techniques, such as summarising and feeding back an interviewee’s account, and using self-disclosure techniques whereby the interviewer asks questions that elicit personal information from an interviewee, as well as providing personal information about themselves [32]. These verbal and non-verbal techniques are also adopted by humanitarian interviewing, which is another rapport-based interviewing model [38]. This model further highlights empathy as key to establishing rapport, whereby an interviewer expresses understanding for the interviewee’s situation through supportive utterances and gestures.

The verbal and non-verbal rapport techniques indicated by the Tickle-Degnen and Rosenthal model and humanitarian interviewing are well operationalised. Indeed, many are key elements of forensic interview frameworks and guidance documents such as the cognitive interview [23], the UK interview model [29], and achieving best evidence [27]. Unsurprisingly therefore, police report regularly using verbal and non-verbal rapport-building techniques with witnesses and they perceive them to be effective [14, 49], albeit perceptions may not necessarily reflect actual behaviour [1, 49].

### Experimental manipulations of rapport

A recent systematic review of the literature base has found that only a small number of studies have experimentally manipulated the use of verbal and non-verbal rapport-building techniques with adults in mock witness settings [15]. Collins and colleagues found that participants interviewed when rapport was present were more positive about the interview experience and recalled more correct information [16]. Here, the interviewer expressed positivity and was attentive, primarily using non-verbal behaviours such as adopting open and relaxed body language and a gentle tone of voice. In the no-rapport conditions, the interviewer was forceful and abrupt, and showed a lack of interest, or neutral in all behaviours. Others have used primarily verbal rapport-building techniques such as the use of self-disclosure questions and prompts (e.g., tell me about your family), and the discussion of personal information prior to the interview [20, 39]. Again, participants had more positive perceptions and improved memory performance in the verbal rapport conditions versus when verbal rapport was absent.

Holmberg and Madsen tested the humanitarian interview, which uses a combination of verbal and non-verbal rapport-building techniques. Again, participants had more positive perceptions of the interviewer and recalled more details of an experienced event than those in the no-rapport interview condition where the interviewer was indifferent, unemotional and unfriendly [38]. However, other studies have reported that verbal rapport building increased the amount of incorrect information recalled by mock witnesses [18], and that a combination of verbal and non-verbal techniques had no impact at all on adult mock witness memory performance [40]. As such, the current literature is mixed regarding the impact of rapport.

There are also several limitations in this empirical literature that makes understanding the true impact of rapport difficult, and the following concerns are echoed by Gabbert and colleagues in their recent review [15]. Firstly, the current research is dominated by investigations of verbal rapport with little focus on non-verbal behaviours, but it is often unclear whether researchers have truly distinguished between verbal and non-verbal behaviours when manipulating rapport. For example, studies that investigated the effect of verbal rapport-building techniques, such as self-disclosure, also reported using active listening techniques which are often accompanied by non-verbal behaviours (e.g., head nodding, eye contact) aimed at expressing interest and attention towards the interviewee and what they had to say, which makes it impossible to interpret the locus of effect (i.e., verbal or non-verbal techniques, or a combination; [18, 20, 39]). Only by isolating verbal and non-verbal rapport behaviours can we begin to understand which behaviours are more effective for building rapport in witness interviews, and the impact each has on memory performance [14, 15].

Secondly, rapport-building techniques are often described in a vague manner, such as the interviewer using friendly behaviours [16] or engaging in a personal conversation with the interviewee [38]. Consequently, these techniques are difficult to operationalise and so the research cannot be experimentally replicated. Thirdly, rapport is measured inconsistently, with some studies having participants write a sentence describing their perception of the interviewer [16] while others ask participants to rate their experiences using different lists of characteristics related to rapport [18, 20, 38–40]. There is also little precedent for analysing interviewer behaviours to ensure adherence to rapport protocols, and so interviewers’ use of rapport and interviewees’ perceptions of rapport across studies cannot be directly compared. Finally, many experimental studies have used interviewing methods that do not reflect UK interviewing practice, such as getting participants to provide a written rather than verbal account [16] or incorporating pre-prepared cued or closed-choice questions [18, 20, 38–40], which can also have detrimental effects on memory recall [50, 51].

### The present study

Increased interest in rapport-based interviewing highlights a need for further research, yet the number of published experimental rapport-building studies with adults is small, rapport is typically poorly operationalised, and verbal and non-verbal behaviours are not clearly separated. As a first step towards filling this gap in understanding, we use the traditional mock-witness paradigm comprising an interview procedure based on the UK investigative interviewing model to investigate verbal and non-verbal rapport-building techniques on episodic memory performance. A clearly operationalised cluster of verbal and non-verbal techniques were manipulated (present or absent) and interviewee’s perceptions were collected immediately post interview.

We drew on the prevalent empirical and theoretical rapport-building literature (e.g., [4, 14, 16, 18, 20, 32, 38–40]), as well as practical guidelines recommended for use by UK police officers (e.g., [23, 27, 29]) to guide the types of rapport behaviours used–the chosen behaviours have also recently been found to be those most commonly used in previous rapport research [15], lending further support for their use. Verbal techniques included interviewer and interviewee self-disclosure, verbal active listening techniques (e.g., summarisation) and empathic utterances. Non-verbal techniques included smiling, nodding, adopting a relaxed posture and body language, and making eye contact (see interview protocols for full details). Given the clear paucity of relevant experimental literature we did not hypothesise, instead preferring to investigate the following broad research questions: are some types of rapport building better received than others, and what is the impact of different types of rapport building on episodic recall during an investigative interview?

## Materials and methods

### Ethics

This research was approved by the University of Westminster ethics committee (ETH1819-0021) and adhered to the British Psychological Society’s (BPS) code of conduct. Data was handled in line with the Data Protection Act and GDPR guidelines. Informed consent was acquired prior to participation, and a debrief of study manipulation was provided at the end of the study.

### Participants

Eighty adults participated in this study, recruited through convenience sampling whereby we advertised the research widely to recruit participants between the ages of 18 and 50 years of age. The sample comprised 68 women and 12 men, ranging in age from 18 to 47 (*M* = 23.37, *SD* = 6.14). All were unknown to the interviewer and lived in the UK. Participants were randomly allocated to one of the four experimental conditions on a first come first served basis (described in the interview protocols section). Convenience sampling was used since i) this was the easiest way to recruit participants for this study at that time, and ii) this mirrors, to some extent, the experiences of non-specialist police investigators who interview non-vulnerable populations. That is, they do not/cannot select at random whom they interview. Given our sampling approach, clearly not all adults between the ages of 18 and 50 had an equal chance of taking part in this research. Therefore, despite the broad age range and that we did not include any prescriptive cognitive inclusion/exclusion assessments (in line with most research in the domain of applied cognition), the extent to which our findings can be generalised to the entire adult population should be considered when interpreting our results (see [52, 53]).

An a-priori G*Power analysis [54] was conducted to calculate the sample size for this research. A literature search for similar relevant research in the domain of applied forensic cognition and rapport building guided the effect size chosen for the G*Power calculation (see [55]). Few experimental papers have been published, however based on the effect sizes reported by other studies (see [15] for an overview of these studies) it is sensible to expect a medium to large effect. Accordingly, G*Power calculations indicated a sample of 80 participants was adequate to detect medium-large main and interaction effects (Cohen’s *f* = .32, assuming power = .80 and *a* = .05). This sample size offers a ‘per cell’ sample that is at least equivalent to much of the published experimental rapport-building research in interview contexts, and in some cases larger (again, see [15] for a review of this research).

### Mock crime stimulus video

Participants first watched a pre-recorded video of a mock crime in conditions of intentional encoding. The video was approximately 1 minute and 40 seconds and depicted a fight between two men in a bar. It was filmed from the first-person perspective of a woman who meets a male friend in the bar and then witnesses an argument between two men which culminates in a fight and an assault where one of the men is knocked unconscious.

### Post-interview perceptions questionnaire

All participants completed a post-interview questionnaire asking them to rate their perceptions of the interviewer and how they felt about their interview experience on multiple characteristics related to rapport. For example, how friendly they found the interviewer or how cooperative they felt the interview to be–while there is currently no consistently used measure of rapport in the literature, this questionnaire was based on one used in several other experimental rapport studies (see [17, 20, 56]). The questionnaire comprised 10 questions, 5 related to perceptions of the interviewer and 5 related to perceptions of the interview process (see S1 Appendix), each of which was rated on a 5-point Likert scale (1 = not at all to 5 = extremely).

### Interview protocols

We developed four distinct interview protocols for this research (described in full below). However, every interview (irrespective of condition) was similarly structured and followed the UK PEACE model (see S2 Appendix), and all included the same four ground rules in the following order, and presented at the same stage of the interview (verbatim): i) ‘please provide *all* the information you can remember in as much detail as possible’, ii) ‘please do not guess information, I only want you to tell me what you actually remember’ iii) ‘please tell me partial and incomplete pieces of information’, and iv) ‘please ask me to repeat any questions that you did not hear or understand’.

Following the ground rules, all interviews commenced with a free recall phase where participants were asked to recall everything they remember from the video. Following the end of their response, the interviewer prompted for more information by asking, ‘Is there anything else you can recall from the video?’. During the free recall account, the interviewer took bullet point notes of the main topics recalled by the interviewee in the order they were recalled. Once the free recall phase had ended, the ground rules were repeated and then the interviewer asked open-ended probing questions about each of the major topics verbalised during the free recall phase, in turn. For example, ‘You recalled seeing a man, so please think back to the film and tell me everything you remember about him’. If a major detail was present in the video but not mentioned by participants in their free recall account, then they were not asked about this.

After every topic had been probed, the interviewer asked if there were any additions or alterations participants would like to make to their account. The interview was then complete. Interview protocols differed across the four conditions only as a function of the presence or absence of rapport, and the type of rapport employed by the interviewer (see below). To reduce the confounding impact of interviewer variability, particularly since this research concerns physical behaviour and verbal behaviour, the same male interviewer conducted all interviews.

#### Verbal rapport (only)

The interviewer engaged in a 5-minute verbal only rapport-building phase prior to explaining the ground rules (see S2 Appendix). Here the interviewer displayed six distinct verbal techniques (see Table 1). Techniques 1 to 4 were used from the offset, whereas techniques 5 and 6 were used where appropriate (i.e., technique 6 only when the interviewee’s name became known and after the interviewee agreed to be referred to by his/her preferred name, and technique 5 from the end of the free recall and then through to the end of the interview). The interviewer’s non-verbal behaviour was identical to the control condition (see below).

**Table 1:** Verbal and behavioural rapport-building techniques.

**Verbal Techniques**	
**1.**	Evocative prompts (e.g, “How are you today?”; “How are you feeling about the interview?”)
**2.**	Questions/prompts to elicit self-disclosure (e.g., “Where are you from”’; “Tell me about your job”)
**3.**	Interviewer self-disclosure (e.g., “I’m from xxxx”; “I’m working here at the University as a researcher”)
**4.**	Comforting/empathic statements (e.g., “I want to assure you that I’m going to be patient and give you as much time as you need to recall the video scene”)
**5.**	Summarises interviewee responses (e.g., “So you remember seeing a white man with short blonde hair and wearing dark blue jeans. Is that correct?”)
**6.**	Refers to interviewee by name
**Behavioural Techniques**	
**1.**	Greets interviewee with a handshake
**2.**	Sits relaxed (i.e., both arms on the table and body leaning towards the interviewee)
**3.**	Uses hand gestures when speaking
**4.**	Maintains frequent eye contact
**5.**	Uses a range of facial expressions (e.g., smiling)
**6.**	Nods and utters "mhm" when listening
**7.**	Speaks in a dynamic tone of voice

#### Behavioural rapport (only)

Here, all 7 behavioural techniques (see Table 1) were employed immediately upon arrival of the interviewee to the interview room and were continued throughout the interview, as appropriate. The interviewer exhibited none of the verbal techniques.

#### Full rapport (behavioural + verbal rapport)

The interviewer engaged in a 5-minute rapport-building phase prior to explaining the ground rules that included both verbal and behavioural techniques (see Table 1; see S2 Appendix). As in the verbal only condition (see above), techniques 1 to 4 were used from the offset, whereas techniques 5 and 6 were used where appropriate. As was the case in the behavioural only condition, all 7 behavioural techniques (see Table 1) were employed from the offset and were combined with the verbal techniques as appropriate throughout the interview.

#### Control (no rapport)

The interviewer did not exhibit any of the behavioural or verbal rapport-building techniques at all throughout the interview. Here, the interviewer did not shake the participant’s hand, sat still and in an upright position, spoke monotonously, made little to no eye contact, facial expressions, hand gestures or nodding, and exhibited none of the verbal techniques.

### Procedure

First, participants were sent a one-time link to the stimulus video 24-hours prior to the interview, which they watched remotely. The link was active for one hour and immediately became inactive after the participant had watched the video. After watching the video, participants were randomly allocated to one of four interview conditions and interviewed 24-hours later. Upon arrival for the interview, the interviewer immediately displayed condition-appropriate behaviours as described in the interview protocols–for example, in the behavioural and full rapport conditions, participants would be met with a handshake, whereas this was not done in the control and verbal rapport conditions. Once the participant had sat down, the video camera and the digital audio recorder were turned on and the interview commenced. All interviews were audio recorded, and the interviewer (only) was video recorded to ensure compliance with the rapport manipulations and interview protocols across conditions. Once the interview was complete, the participant completed the rapport perceptions questionnaire in the absence of the interviewer.

#### Memory performance coding

Interviews were transcribed verbatim and coded for the number of correct, erroneous (e.g., reporting that the man’s bag was black, when in fact it was brown), and confabulated information items (reporting a detail or event that was not present or did not happen) verbalised from the commencement of the *free recall* phase until the end of the *questioning* phase. The position within the interview that the information was verbalised was noted (free recall or questioning), and information items were only coded once (on first mention). The percentage accuracy of the information reported was also calculated by dividing the number of correct items reported by the total number of items reported (the sum of correct information, errors and confabulations).

The first author (ZN) coded all interviews. Sixteen interviews (four from each condition) were selected at random and double-coded independently by a research assistant who was naïve to the research questions and the experimental conditions. This accounted for 20% of the interviews. Intraclass correlation coefficient (ICC) analyses, testing for absolute agreement between the first author (ZN) and the research assistant using a two-way random model were conducted on the three performance measures and percentage accuracy. ICC indicated good inter-rater reliability for all three measures: total correct, R_2_ = .979, df = 15, 15, *p* < .001; total errors, R_2_ = .867, df = 15, 15, *p* < .001; total confabulations, R_2_ = .911, df = 15, 15, *p* < .001. ICC also indicated good inter-rater reliability for percentage accuracy, R_2_ = .892, df = 15, 15, *p* < .001.

#### Interviewer behaviour coding

A random selection of 4 interviews from each condition (16 in total, 20% of the sample) were analysed for interviewer behaviour. The literature pertaining to the coding of rapport behaviours by interviewers is scant, and as far as we are aware there is very little precedent for coding rapport (but see [57, 58]). In accordance with empirical interviewing research on the presence/absence of behaviours (e.g., [59, 60]) and with reference to Johnston and colleagues [58], we developed a study specific objective rapport coding scheme, as follows. First, each of the 13 rapport variables (6 verbal & 7 behavioural) were listed and fully described on a coding sheet. Second, coders were offered 3 scoring categories for each of the 13 variables listed and described. The categories were absent, partially present, or fully present (absent = 0; partially present = 1; fully present = 2). As the verbal and full rapport conditions (only) incorporated a 5-minute verbal rapport-building phase at the start of the interview, the interviews were sectioned (sliced) and coded for interviewer behaviours in both the rapport-building phase and the interviewing phase (including both the free recall and questioning phases of the interview).

The 16 interviews were individually scored by two independent coders who were naïve to the research questions as follows: to score 0 the variable in question had to be completely absent, to score 1 the variable had to be present at least once and no more than twice in the relevant phase, whereas to award 2 the variable had to be present at least 3 times or more in that. Prior to coding the coders participated in a training session held by the second author (CD) during which they practiced coding and discussed any disagreements/misunderstandings to reach a consensus. Intraclass correlation coefficient (ICC) analyses, testing for absolute agreement between the two coders and using a two-way random model were conducted on the coding. ICC’s indicated good inter-rater reliability for both behavioural, R_2_ = 1, df = 27, 27, *p* < .001, and verbal techniques, R_2_ = .998, df = 23, 23, *p* < .001. The mean scores (for both coders) for each verbal and behavioural technique across interviews as a function of the (relevant) interview phases are displayed in Tables 2–4 (note the behavioural condition did not include a verbal rapport-building phase). The scores for the control condition are not presented in a table or included in the following analyses as every verbal and behavioural rapport technique was coded as absent by each coder, all *Ms* = 0 (*SDs* = 0).

**Table 2:** Mean coding and Kruskal-Wallis rankings (standard deviations) for techniques in the verbal rapport condition as a function of the rapport-building and interviewing phase.

	Rapport-building phase		Interviewing phase	
	M (SD)	M_rank_	M (SD)	M_rank_
**Verbal techniques**				
Evocative prompts	1.75 (.50)	4.00	1.75 (.29)	10.50
Eliciting self-disclosure	1.00 (0)	2.50	.06 (.13)	7.50
Interviewer self-disclosure	1.13 (.25)	2.63	0 (0)	6.50
Comforting/empathic statements	1.13 (.25)	3.13	.75 (.29)	9.25
Summarising	0 (0)	4.50	1.57 (.13)	9.00
Using names	1.00 (0)	4.50	2.00 (0)	10.50
**Behavioural techniques**				
Handshaking	0 (0)	2.50	0 (0)	5.50
Relaxed posture	0 (0)	2.50	0 (0)	2.50
Hand gestures	0.13 (.25)	2.50	.13 (.14)	2.50
Eye contact	0.38 (.48)	2.50	0 (0)	2.50
Facial expressions	0 (0)	2.50	0 (0)	2.50
Nodding	0.38 (.48)	2.50	.13 (.14)	2.50
Dynamic tone	0 (0)	2.50	0 (0)	2.50

**Table 3:** Mean coding and Kruskal-Wallis rankings (standard deviations) for the techniques in the behavioural rapport condition.

	Interviewing phase	
	M (SD)	M_rank_
**Verbal techniques**		
Evocative prompts	0 (0)	3.00
Eliciting self-disclosure	0 (0)	6.00
Interviewer self-disclosure	0 (0)	6.50
Comforting/empathic statements	0 (0)	2.50
Summarising	0 (0)	2.50
Using names	0 (0)	2.50
**Behavioural techniques**		
Handshaking	.13 (.25)	7.00
Relaxed posture	2.00 (0)	8.50
Hand gestures	2.00 (0)	8.50
Eye contact	2.00 (0)	8.50
Facial expressions	2.00 (0)	8.50
Nodding	2.00 (0)	8.50
Dynamic tone	2.00 (0)	8.50

**Table 4:** Mean coding and Kruskal-Wallis rankings (standard deviations) for the techniques in the full rapport condition as a function of the rapport-building and interviewing phase.

	Rapport-building phase		Interviewing phase	
	M (SD)	M_rank_	M (SD)	M_rank_
**Verbal techniques**				
Evocative prompts	2.00 (0)	5.00	0.38 (.25)	6.00
Eliciting self-disclosure	2.00 (0)	6.50	0 (0)	6.00
Interviewer self-disclosure	1.88 (.25)	6.38	0 (0)	6.50
Comforting/empathic statements	1.75 (.50)	5.88	.56 (31)	7.75
Summarising	0 (0)	4.50	1.50 (0)	8.00
Using names	1.00 (0)	4.50	1.00 (0)	6.50
**Behavioural techniques**				
Handshaking	1.00 (0)	6.50	.13 (.25)	7.00
Relaxed posture	2.00 (0)	6.50	2.00 (0)	8.50
Hand gestures	2.00 (0)	6.50	2.00 (0)	8.50
Eye contact	2.00 (0)	6.50	2.00 (0)	8.50
Facial expressions	2.00 (0)	6.50	2.00 (0)	8.50
Nodding	2.00 (0)	6.50	2.00 (0)	8.50
Dynamic tone	2.00 (0)	6.50	2.00 (0)	8.50

#### Manipulation analysis

A series of Kruskal-Wallis tests were conducted to investigate our manipulation of the presence/absence of the six verbal and seven behavioural rapport techniques across conditions as a function of phase, followed by Mann-Whitney post-hoc tests, as appropriate.

The verbal and full rapport conditions were the only conditions which comprised a rapport-building phase, and so were the only conditions included in the analysis for the rapport-building phase. Eliciting self-disclosure from the interviewee and interviewer self-disclosures occurred more frequently in the full condition than in the verbal condition, *H*(1) = 6.40, *p* = .011, and, *H*(1) = 5.25, *p* = .022, respectively. The remaining four verbal behaviours (see Table 1) did not significantly differ between the verbal and full rapport conditions, all *p*s > .05. All seven behavioural rapport techniques were displayed significantly more frequently in the full condition than in the verbal condition, all *Hs*(1) < = 7.00, all *ps <* = .013.

For verbal rapport techniques in the interviewing phase of the verbal, behavioural and full conditions, overall the occurrence of evocative prompts, comforting/empathic statements, summarising responses and using the interviewee’s name differed significantly, all *Hs* (2) < = 7.00, all *p*s < = .013. Post-hoc tests revealed that all four techniques occurred more often in the verbal rapport condition than in the behavioural rapport condition, all *ps* < = .017. Summarising responses also occurred more frequently in the full rapport compared to the behavioural rapport condition, *p* = .042. Analysis of the seven behavioural techniques in the interviewing phase across the verbal, behavioural and full conditions revealed a significant difference for six of the techniques: relaxed body posture, hand gestures, eye contact, facial expressions, nodding, and dynamic tone of voice, all *Hs*(2) < = 7.00, all *ps* < = .013. Each technique was more common in the behavioural and full conditions than in the verbal condition, all *ps <* = .015. No differences emerged for handshaking in the interviewing phase, all *p*s > .05.

To summarise, all behavioural rapport techniques were more prominent in the appropriate conditions (i.e., the behavioural and full rapport conditions) compared to the verbal rapport condition, where they were mostly absent (see Table 2). All verbal rapport techniques were present in the rapport-building phase of the verbal and full rapport conditions (apart from summarisation, which is a technique more relevant to the interviewing phase), as well as in the interviewing phase of these conditions (apart from self-disclosure techniques, which are more suitable to the rapport-building phase). Our analyses showed that they were generally more prominent in these conditions compared to the behavioural rapport condition (see Tables 2–4). Verbal techniques were more difficult to control than behavioural techniques, with our analyses showing that some verbal techniques were presented to varying degrees between the verbal and full rapport conditions, but were present to some extent in both conditions while being completely absent in the behavioural rapport condition (see Table 3), and so our manipulations were successful in bringing about differences in rapport behaviours across conditions.

## Results

### Memory analysis approach

We conducted a series of 2 (verbal rapport: present, absent) x 2 (behavioural rapport: present, absent) ANOVAs to investigate main effects and interactions on overall (global) memory performance for the amount of correct, incorrect (errors), and confabulated details, and percentage accuracy. Analysis of global episodic performance alone provides no information about the impact, or otherwise, of rapport on the ‘parts’ of the interview and whether they contribute to global performance. Thus, in order to fully understand the locus of any effects we also analysed performance as a function of interview phase (i.e., free and probed recall).

#### Overall (global) memory performance

A significant main effect of behavioural rapport emerged for the amount of correct information reported, *F*(1,76) = 10.02, *p* = .002, η_p_^2^ = .12. When behavioural rapport was present significantly more correct event details were reported than when it was absent (see Table 5). All other main effects and interactions were non-significant for correct recall, all *Fs* < = 0.78, all *ps* > .05.

**Table 5:** Mean (standard deviations [95% confidence intervals]) main effects and interactions for global correct information, incorrect information (errors), confabulations (confabs) and accuracy (* = *p* < .05).

	Behavioural Rapport		Verbal Rapport		Behavioural + Verbal Rapport	
	Present	Absent	Present	Absent	Present	Absent
Global Performance	M (SD) [95% CI]					
Correct	*69.90 (12.41) [65.93; 73.87]	*61.53 (11.03) [58.00; 65.05]	64.55 (12.49) [60.55; 68.55]	66.88 (12.36) [62.92; 70.83]	67.23 (12.66) [64.41; 70.04]	64.20 (13.61) [61.54; 66.86]
Errors	15.68 (6.58) [13.58; 17.77]	14.10 (6.38) [12.06; 16.14]	16.20 (7.49) [13.80; 18.60]	13.58 (5.03) [11.97; 15.18]	15.94 (7.00) [14.38; 17.50]	13.84 (5.71) [12.57; 15.11]
Confabs	5.85 (3.48) [4.74; 6.96]	6.33 (5.47) [4.57; 8.08]	6.10 (4.94) [4.52; 7.68]	6.08 (4.21) [4.73; 7.42]	5.98 (4.25) [5.03; 6.92]	6.20 (4.85) [5.12; 7.28]
Accuracy (%)	77.27 (6.38) [75.23; 79.31]	76.37 (7.34) [73.90; 78.85]	75.54 (7.45) [73.16; 77.93]	78.10 (6.48) [76.02; 80.17]	76.40 (6.95) [74.86; 77.95]	77.24 (7.15) [75.65; 78.83]

There were no significant main effects nor interactions for the overall amount of incorrect or confabulated details reported, all *Fs* < = 3.35, all *ps* > .05 (see Table 5 for main effect and interaction means, SDs and 95% CIs). Analysis of overall percentage accuracy also revealed no significant main effects nor interactions, all *Fs <* = 2.64, all *ps >* .05.

#### Interview phase memory performance

Interviews comprised two distinct phases, namely free and probed recall. Analysis of correct information, errors, confabulations and percentage accuracy as a function of phase (see Table 6 for all means, SDs, & 95% CIs) revealed a significant main effect of behavioural rapport for the amount of correct information recalled in the free recall phase, *F*(1,76) = 4.13, *p* = .046, η_p_^2^ = .051. More correct information was reported when behavioural rapport was present compared to absent. A significant main effect of verbal rapport was also found for percentage accuracy in the free recall phase, *F*(1,76) = 7.84, *p* = .006, η_p_^2^ = .094. Participants were significantly less accurate when verbal rapport was present compared to absent. All other main effects and interactions were non-significant in the free recall phase, all *F*s < = 2.84, all *p*s > .05.

**Table 6:** Mean (standard deviations [95% confidence intervals]) main effects and interactions for correct information, incorrect information (errors), confabulations (confabs) and accuracy across recall phases (* = *p* < .05).

	Behavioural Rapport		Verbal Rapport		Behavioural + Verbal Rapport	
	Present	Absent	Present	Absent	Present	Absent
	M (SD) [95% CI]					
**Free Recall**						
Correct	*38.45 (12.13) [34.57; 42.33]	*33.68 (8.54) [30.94; 36.41]	34.58 (11.61) [30.86; 38.29]	37.55 (9.61) [34.48; 40.62]	36.51 (11.96) [33.85; 39.17]	35.61 (9.24) [33.56; 37.67]
Errors	4.50 (3.78) [3.29; 5.71]	3.80 (2.15) [3.11; 4.49]	4.73 (3.87) [3.49; 5.96]	3.58 (1.88) [2.97; 4.18]	4.61 (3.80) [3.77; 5.46]	3.69 (2.01) [3.24; 4.13]
Confabs	2.00 (1.87) [1.40; 2.60]	1.85 (1.81) [1.27; 2.43]	2.20 (1.76) [1.64; 2.76]	1.65 (1.87) [1.05; 2.25]	2.10 (1.80) [1.70; 2.50]	1.75 (1.83) [1.34; 2.16]
Accuracy (%)	86.10 (7.27) [83.78; 88.43]	86.05 (7.05) [83.80; 88.31]	*83.93 (8.18) [81.31; 86.54]	*88.23 (5.10) [86.60; 89.87]	85.0 (7.77) [83.28; 86.74]	87.14 (6.21) [85.76; 88.53]
**Probed Recall**						
Correct	*31.73 (6.68) [29.59; 33.86]	*27.85 (6.70) [25.71; 29.99]	30.23 (6.91) [28.01; 32.44]	29.35 (7.01) [27.11; 31.59]	30.98 (6.80) [29.46; 32.49]	28.60 (6.86) [27.07; 30.13]
Errors	11.25 (5.07) [9.63; 12.87]	10.30 (5.14) [8.66; 11.94]	11.58 (5.61) [9.78; 13.37]	9.98 (4.46) [8.55; 11.40]	11.41 (5.31) [10.23; 12.60]	10.14 (4.78) [9.07; 11.20]
Confabs	3.85 (2.43) [3.07; 4.63]	4.48 (4.43) [3.06; 5.89]	3.88 (3.96) [2.61; 5.14]	4.45 (3.15) [3.44; 5.46]	3.86 (3.26) [3.14; 4.59]	4.46 (3.82) [3.61; 5.31]
Accuracy (%)	68.81 (9.28) [65.84; 71.77]	65.79 (12.44) [61.82; 69.77]	67.83 (10.66) [64.42; 71.24]	66.77 (11.46) [63.11; 70.44]	68.32 (9.94) [66.11; 70.53]	66.28 (11.90) [63.64; 68.93]

A significant main effect of behavioural rapport also emerged for the amount of correct information recalled in the probed recall phase, *F*(1,76) = 6.69, *p* = .012, η_p_^2^ = .081. Again, more correct information was reported when behavioural rapport was present than when it was absent (see Table 6). All other main effects and interactions were non-significant in the probed recall phase, all *F*s < = 1.97, all *p*s > .05.

### Post-interview perceptions

Multivariate analysis of variance (MANOVA) on participants’ post-interview perceptions of the interviewer (grouping these 5 items) and interview process (grouping these 5 items) revealed significant multivariate effects of condition for perceptions of the interviewer, *F*(15,199.16) = 5.90, *p* < .001, Wilks’ Lambda = .36, η_p_^2^ = .29, and the interview process, *F*(15,199.16) = 4.84, *p* < .001, Wilks’ Lambda = .42, η_p_^2^ = .25, (see Table 7 below). Univariate analyses, applying Bonferroni corrected alphas of .005 to correct for 10 comparisons between 5 items in each subscale, revealed significant differences across conditions for all 5 interviewer and 5 interview process perception ratings (see Table 7 for ANOVA results, means, SDs, & 95% CIs).

**Table 7:** Mean post-interview perceptions (standard deviations [95% confidence intervals]) and univariate results across conditions.

	Condition						
	Control	Verbal	Behavioural	Full			
	M (SD) [95% CI]				*F*(3,76)	*p*	η_p_^2^
**Interviewer**							
Q1. Interviewer was friendly	2.25 (1.07) [1.75; 2.75]	2.60 (1.23) [2.02; 3.18]	4.25 (.91) [3.82; 4.68]	4.45 (.69) [4.13; 4.77]	25.50	< .001	.50
Q1. Interviewer was awkward	2.25 (1.12) [1.73; 2.77]	2.65 (1.42) [1.98; 3.32]	1.05 (.22) [.95; 1.15]	1.25 (.72) [.91; 1.59]	12.42	< .001	.33
Q3. Interviewer was bored	1.75 (1.07) [1.25; 2.25]	2.40 (1.31) [1.79; 3.01]	1.30 (.66) [.99; 1.61]	1.25 (.55) [.99; 1.51]	6.31	.001	.20
Q4. Interviewer was attentive	3.60 (1.23) [3.02; 4.18]	3.80 (1.11) [3.28; 4.32]	4.65 (.59) [4.38; 4.92]	4.55 (.69) [4.23; 4.87]	6.27	.001	.20
Q5. Interviewer was respectful	4.05 (.76) [3.69; 4.41]	3.90 (1.21) [3.33; 4.47]	4.40 (.60) [4.12; 4.68]	4.80 (.41) [4.61; 4.99]	5.01	.003	.17
**Interaction**							
Q6. Interview was cold	2.85 (1.35) [2.22; 3.48]	3.35 (1.53) [2.63; 4.07]	1.25 (.55) [.99; 1.51]	1.35 (.67) [1.04; 1.66]	18.28	< .001	.42
Q7. Interview was positive	2.25 (1.07) [1.75; 2.75]	2.35 (1.09) [1.84; 2.86]	3.55 (1.10) [3.04; 4.06]	4.15 (.99) [3.69; 4.61]	15.28	< .001	.38
Q8. Interview was comfortably paced	3.40 (1.31) [2.79; 4.01]	2.75 (1.29) [2.15; 3.35]	4.15 (.75) [3.80; 4.50]	4.30 (.80) [3.92; 4.68]	8.97	< .001	.26
Q9. Interview was cooperative	3.20 (.83) [2.81; 3.59]	3.05 (1.36) [2.42; 3.68]	3.80 (.62) [3.51; 4.09]	4.30 (.57) 4.03; 4.57]	8.17	< .001	.24
Q10. Interview was engaging	2.85 (.81) [2.47; 3.23]	2.65 (1.23) [2.08; 3.22]	3.75 (1.12) [3.23; 4.27]	3.80 (1.06) [3.31; 4.29]	6.31	.001	.20

A series of Games-Howell post-hoc tests revealed no differences for any of the interviewer or interview process perceptions between the control and verbal conditions, nor between the full and behavioural conditions, all *p*s > .05. Participants in the control (no rapport) condition reported the interviewer as significantly more awkward, less attentive, and less friendly, and found the interview process more cold, less engaging and less positive than participants in both the full and behavioural (only) conditions. They also perceived the interviewer to be less respectful and the interview process less cooperative in nature than in the full condition, all *ps* < = .03.

Participants in the verbal (only) condition rated the interviewer as appearing significantly more bored, more awkward, and less friendly, and found the interview process more cold, less engaging, less positive, and the pace of the interview far less comfortable than participants in both the behavioural (only) and full rapport conditions. Participants in the verbal (only) condition also found the interviewer less attentive and found the interview process to be less cooperative than those in the full rapport condition, all *p*s < = .026.

## Discussion

We empirically investigated the impact of clusters of different types of rapport-building behaviours on witness memory at retrieval. We also considered which clusters were best received by mock witnesses by collecting post-interview feedback. The rationale for this research was twofold. First, the empirical literature is dominated by post-hoc investigations of rapport building (e.g., presence, absence, and likely impact) by accessing recordings or transcripts of real life interviews with witnesses and persons of interest [11, 22, 25, 26, 28, 36, 37], with relatively little attention paid to the impact of rapport behaviours on episodic memory performance. Second, because of the limited nature of experimental work that does consider episodic memory (e.g., verbal and behavioural techniques are often not separated), the locus of effect for rapport is far from clear and the results are somewhat contradictory (e.g., [16, 18, 20, 38–40]). Hence, it is difficult to understand the true impact of rapport on memory performance and whether, through an interpersonal lens, witnesses ‘feel’ any experiential differences.

Our primary finding was that behavioural rapport was well received by mock witnesses, whereby they recalled 12% more correct event information without a concomitant increase in errors or confabulations when the interviewer expressed behavioural rapport compared to when behavioural rapport was absent. Moreover, this improved memory performance emerged across both interview phases (i.e., free and probed recall), indicating a robust effect. Despite the improved memory performance for behavioural rapport, percentage accuracy was not statistically significant. Here, accuracy differences overall and as a function of phase were too small to trigger significant findings, a pattern of accuracy results that are not unusual in applied research of this nature (e.g., see [61–63]).

Our findings also revealed that verbal rapport building alone had a negative impact on the accuracy of the information recalled, which dropped significantly in the initial free recall phase, and post-interview feedback indicates that verbalisations alone were poorly received. These findings suggest that scripted verbal rapport without the physical behaviours that one might expect to accompany rapport-building verbalisations has the potential to hinder the rapport-building process and witness memory performance. That this drop in memory performance was only found in the initial phase of the interview also suggests that interviewees may have initially been uncomfortable but then perhaps became accustomed to this behaviour as the interview progressed.

### Implications of verbal and non-verbal rapport-building behaviours

Non-verbal behaviour is often argued as being one of the most powerful methods of communication [45, 64–66], and research in other domains has long championed the suggestion that ‘actions speak louder than words’ (e.g., [67–69]). In goal-dependant contexts such as an interview, where interviewers are seeking to manage the social context to support cognition, the perception–behaviour link is well established (see [70, 71] for reviews). Non-verbal communication may effectively support the interviewer’s goal–episodic retrieval–because information about a social ‘relationship’ can be easier, and more automatically and unconsciously inferred from behaviour, thus reducing demands on limited cognitive resources. Indeed, research concerning conscious and unconscious behavioural mimicry has indicated increased liking and more comfortable interactions in general [72, 73], which has often resulted in improved goal attainment [74–76].

Our results lend support for these arguments, and in doing so extend the literature on rapport and eyewitness memory by providing promising evidence of a quantifiable, positive impact of a cluster of pro-social behaviours on cognition in a witness interview. The post-interview perceptions data revealed that participants’ experiences were far more positive when supportive social rapport-building behaviours were exhibited, with the interviewer rated as far warmer, more engaging, and more friendly by participants in the behavioural (only) rapport condition compared to those in the verbal (only) rapport and control (no rapport) conditions. They also reported finding the pace of the interview more comfortable. As with the memory performance results, there were no differences in perceptions between the behavioural (only) and full (behavioural + verbal) rapport conditions.

These findings concur with the findings of previous research that has used primarily non-verbal behaviours [16]. However, they differ to the pattern of positive results reported by others (e.g., [20]) where verbal rapport was well received and found to improve the quality of information by reducing errors–although, previous findings for verbal techniques may have been influenced by the inclusion of *unspecified* positive non-verbal techniques. Conversely, our findings were that verbal techniques alone were poorly received and did not support memory performance, even hindering accurate recall in places. It could be argued that fully separating verbal behaviours from non-verbal behaviours as we did is artificial and not reflective of actual practice. However, verbal behaviours have been found to sometimes be presented with indifference in practice, with some police officers showing little expression or emotion and making their use seem routine or insincere [77, 78]. As such, the verbal rapport condition may represent poor rapport-building practice, and it is possible that in the real world, where witnesses are known to be more anxious and stressed in a manner that is difficult to replicate in the laboratory, paying ‘lip service’ to rapport (that is saying rather than behaving) may negatively impact cognition (e.g., [79, 80]).

Disentangling all of the elements of complex social interactions such as witness interviews to understand the locus of effect is challenging, and given the dearth of research in this domain we are forced to speculate to a certain extent. Our findings for the verbal condition might be explained by the inappropriate use of verbal techniques (i.e., saying things while showing little interest/attention through non-verbal means), such that participants’ experiences were akin to that of participants in the no-rapport condition. It is less clear why full (verbal + behavioural) rapport did not have a significant impact on memory performance. One explanation may be that full rapport building lengthened the pre-retrieval, social phase of interviews, which may have made our mock witnesses more anxious/nervous. Some studies have suggested that extended rapport building can make the interview process more cognitively taxing [40, 81] and may lead to over-rapport, whereby appearing too friendly or familiar risks being perceived as forced or inappropriate when witnesses simply want to ‘get on with it’ [32, 82, 83].

While participants’ perceptions of rapport in the full rapport condition were not negatively affected by the inclusion of verbal techniques, here rapport was only measured post interview. It is possible that if perceptions were also measured after the pre-interview rapport-building phase, a phase not present in the behavioural condition and where the majority of verbal techniques were present, there may have been some differences compared to post interview. The use of verbal techniques may have been perceived as inappropriate or uncomfortable, thereby limiting recall to a degree and resulting in no significant improvement in memory performance when full rapport was present. Behavioural techniques during the interview, however, may have improved perceptions post interview. As such, future research should investigate changes in rapport over the course of an interview, and the effects of over-rapport.

### Limitations and future directions

The present study adds to the limited experimental literature on rapport building and has implications for forensic practice and interview training in general. The strengths of this research are that we used the well-established mock-witness paradigm and controlled interviewer variability. Further, rapport behaviours were clearly operationalised and controlled so as to ensure clear differences across conditions and to allow replication. Verbal and behavioural rapport were also separated so as not to cross contaminate. That said, as with all laboratory studies of this nature, there are limitations, some of which have already been highlighted throughout the discussion.

Arguably, one of the most challenging aspects of this research was controlling the rapport behaviours. Our interviewer behaviour coding and manipulation analysis revealed discernible differences in rapport behaviours, as well as differential experiences by participants across conditions, although some leakage did occur–potentially, difficulty controlling the verbal techniques limited the current study and led to the pattern of findings in the verbal and full rapport conditions. It may also be that our participant self-report measure of rapport was not appropriate. The questionnaire used was chosen due to its use in previous research, but it only measured general perceptions of the interviewer/interaction and not perceptions of specific rapport techniques; perhaps measurement in this way would have yielded different data. These factors reiterate the difficulties of measuring rapport discussed in the introduction, and further consideration of how best to code/measure interviewer rapport-building behaviours and perceptions of rapport is needed. We developed one method, but there is a lack of consensus on how best to measure these.

A further limitation to the current findings is the sample size and population. Participants were relatively homogenous and there was no emphasis placed on gathering a cross-cultural sample. This is a problem common to social science research in general [84], but can impact on the ecological validity of the study. This may be particularly pertinent to the study of rapport. For example, key rapport-building behaviours used in the current study have been shown to be received differently cross-culturally (e.g., eye contact can appear aggressive/disrespectful in East Asian and Middle Eastern cultures [85, 86]). As such, techniques that are frequently used and thought to be effective for building rapport may not generalise to wider and more diverse populations, and future research should aim to tease apart and investigate the plethora of factors that may influence rapport building. Furthermore, future research should also consider using different interviewers, various interview contexts, and employ an unintentional encoding paradigm.

## Conclusion

The clear take home message from this research is that non-verbal behaviours are important for building rapport with adult witnesses–they were well received and positively impacted witness memory performance. Attempts at establishing rapport devoid of non-verbal cues that would commonly accompany verbal communication in an interpersonal context seemed to fail, possibly because they may have appeared disingenuous (see [22] for examples regarding empathy, and see [87] for social cognition and rapport) which risks hindering cognition [32]. When accompanied by non-verbal cues, verbal communication did not significantly impact witness cognition. As such, verbal techniques may not be a necessary component of rapport building, and may provide limited (or no) benefits while also being considerably complex to successfully utilise (as seen by our behavioural analysis). Non-verbal behaviours on the other hand do seem necessary for developing rapport and were simple to use. As such, particularly where time is short, training resources are limited, or interviewers are less experienced, for example, our findings suggest a straightforward way to build rapport through non-verbal behaviours in goal-directed interview settings.

## Supporting information

S1 Appendix Post-interview perceptions questionnaire. (DOCX)

S2 Appendix Interview protocols. (DOCX)

S1 Dataset Memory performance, post-interview perceptions and interviewer behaviour coding data. (SAV)
