# Supplementary material for: Rapport building and witness memory: Actions may ‘speak’ louder than words
Source: PLoS One. 2021 Aug 13;16(8):e0256084. doi: 10.1371/journal.pone.0256084 (PMC8362973; doi:10.1371/journal.pone.0256084)
Supplement: S1 Appendix — (DOCX) [file pone.0256084.s001.docx]

**S1 Appendix. Post-Interview Questionnaire**

Please rate what you thought of the **interviewer** on the following characteristics – **He was:**

| 1. **Friendly** | 1 | 2 | 3 | 4 | 5 |
| --- | --- | --- | --- | --- | --- |
|  | Not friendly | Slightly friendly | Moderately friendly | Very friendly | Extremely friendly |
| 1. **Awkward** | 1 | 2 | 3 | 4 | 5 |
|  | Not awkward | Slightly awkward | Moderately awkward | Very awkward | Extremely awkward |
| 1. **Bored** | 1 | 2 | 3 | 4 | 5 |
|  | Not bored | Slightly bored | Moderately bored | Very bored | Extremely bored |
| 1. **Attentive** | 1 | 2 | 3 | 4 | 5 |
|  | Not attentive | Slightly attentive | Moderately attentive | Very attentive | Extremely attentive |
| 1. **Respectful** | 1 | 2 | 3 | 4 | 5 |
|  | Not respectful | Slightly respectful | Moderately respectful | Very respectful | Extremely respectful |

Please rate what you thought of the **interaction** you had with the interviewer on the following characteristics – **The interaction was:**

| 1. **Cooperative** | 1 | 2 | 3 | 4 | 5 |
| --- | --- | --- | --- | --- | --- |
|  | Not cooperative | Slightly cooperative | Moderately cooperative | Very cooperative | Extremely cooperative |
| 1. **Comfortably Paced** | 1 | 2 | 3 | 4 | 5 |
|  | Not comfortably paced | Slightly comfortably paced | Moderately comfortably paced | Very comfortably paced | Extremely comfortably paced |
| 1. **Cold** | 1 | 2 | 3 | 4 | 5 |
|  | Not cold | Slightly cold | Moderately cold | Very cold | Extremely cold |
| 1. **Engaging** | 1 | 2 | 3 | 4 | 5 |
|  | Not engaging | Slightly engaging | Moderately engaging | Very engaging | Extremely engaging |
| 1. **Positive** | 1 | 2 | 3 | 4 | 5 |
|  | Not positive | Slightly positive | Moderately positive | Very positive | Extremely positive |
