# Supplementary material for: Rapport building and witness memory: Actions may ‘speak’ louder than words
Source: PLoS One. 2021 Aug 13;16(8):e0256084. doi: 10.1371/journal.pone.0256084 (PMC8362973; doi:10.1371/journal.pone.0256084)
Supplement: S2 Appendix — (DOCX) [file pone.0256084.s002.docx]

**S2 Appendix. Interview protocols**

**I. Script for all conditions**

1. **Interviewer** begins with a short introduction:
   1. “Thank you for coming today. My name is xxxx, and I will soon ask you questions related to the video you watched yesterday. Firstly, I want to remind you that you have already consented to this interview being audio and video recorded. Is that still okay with you?
   2. “Do you have any questions before we start the interview?”
2. **Interviewer** sets out the interview ground rules before beginning the interview:
   1. “I want you to tell me everything you can remember for the video in as much detail as possible.”
   2. “I want you only to tell me what you actually remember. Don’t try to guess and please say if you do not know the answer to my question.”
   3. “If you can only remember partial information or small pieces of information, please provide these as they are still valuable.”
   4. “If you don’t understand what I’m asking, please say so and I will try to rephrase the question.**”**
3. Free recall phase begins:
   1. **Interviewer:** “Please can you tell me everything you remember for the video in as much detail as possible.”
   2. At the end, **interviewer** prompts: “Is there anything else you can remember?”
4. **Interviewer** repeats the ground rules (see **I**, step **2**) and then begins the probed questioning phase:
   1. For each of the major topics the participant mentioned in the free recall, the **interviewer** asks: “You mentioned seeing xxxx. Please tell me everything you remember about xxxx.”
   2. Repeat for each topic.
5. **Interviewer** closes the interview:
   1. “Thank you. I think I now have a good idea about what has happened. Just before we finish, is there anything else you want to add or alter?”
6. That is the end of the interview.

**II. Verbal rapport script**

For the verbal and full rapport conditions, prior to the ground rules in the main interview protocol (see **I**, step **2**) the **interviewer** carried out a 5-minute (approximately) rapport-building phase. Here, the **interviewer** followed a semi-structured script using several verbal rapport techniques, such as evocative prompts, self-disclosure, empathic statements and using the interviewee’s name (see below). The **interviewer** also attempted to match the interviewee’s responses in tone and content (see **II**, step **2** for an example).

1. **Interviewer** begins with a short introduction:
   1. “Thank you for coming today xxxx. Is it fine that I call you xxxx? Okay, my name is xxxx, and I am soon going to ask you some questions related to the video that you watched yesterday. Firstly, I want to remind you that you have already consented to this interview being audio and video recorded. Is that still okay with you?”
   2. “Do you have any questions before we start the interview?”
2. **Interviewer** asks: “How are you today xxxx?”
   1. **Interviewer** responds accordingly (e.g., “I’m also doing well/my day has also not been great today”).
3. **Interviewer** asks: “Were you able to find the room okay?”
   1. **Interviewer** responds accordingly (e.g., “okay, good/yeh it can be confusing to find”).
4. **Interviewer** asks about the participant’s experience studying at University (if a student) or in their job (if not a student):
   - 1. **Interviewer** responds by mentioning either their own role as a PhD student (if a student participant) or teaching as an employee at the University (if a non-student participant).
5. **Interviewer** asks the participant about their future plans with their studies/work:
   1. **Interviewer** responds accordingly (e.g., “That sounds like a good plan, I hope it works out”/”I wouldn’t worry too much, it can be difficult to think that far ahead.”)
6. **Interviewer** asks: “Where are you from xxxx?”
   1. **Interviewer** asks about their experience living in London.
   2. **Interviewer** shares where they are from and their experience living in London.
7. **Interviewer** asks: “How was your commute to the University today?”
   1. **Interviewer** responds accordingly (e.g., “oh, that’s quite quick/oh, that’s a long journey”) and shares their own commute.
   2. **Interviewer** says: “I appreciate you making that commute to come and participate in the study today.”
8. **Interviewer** asks: “Have you ever taken part in research before?”
   1. If yes, they are asked about other studies they have participated in.
   2. If no, **interviewer** says: “Okay, no problem. Hopefully you find today’s research interesting and take part in more in the future.”
9. **Interviewer** asks: “How are you feeling about taking part in the interview?”
   1. **Interviewer** says: “I’d just like to assure you that I’m going to be patient and give you as much time as you need to remember the video scene, so try not to worry too much about it.”

During the main interview protocol (see **I**), the **interviewer** also summarised the interviewee’s responses, used the interviewee’s name, and used evocative prompts/empathic statements where appropriate.
